# Inventory of tiger- and ground-beetles (Coleoptera, Caraboidea, Cicindelidae and Carabidae) in two sampling seasons of the Gorongosa National Park, Mozambique

**DOI:** 10.3897/BDJ.11.e101280

**Published:** 2023-08-17

**Authors:** Artur R. M. Serrano, Martim Baptista, Rui Carvalho, Mário Boieiro, Sara Mendes, Marie Bartz, Sérgio Timóteo, Henrique M.V.S. Azevedo-Pereira, Carlos A.S Aguiar, António Alves da Silva, Joana Alves, Maria Jesús I. Briones, Paulo A. V. Borges, José P. Sousa, Pedro Martins da Silva

**Affiliations:** 1 Centre for Ecology, Evolution and Environmental Changes, Faculty of Sciences, University of Lisbon, Rua Ernesto de Vasconcelos Ed. C2, Campo Grande, 1749- 016, Lisbon, Portugal Centre for Ecology, Evolution and Environmental Changes, Faculty of Sciences, University of Lisbon, Rua Ernesto de Vasconcelos Ed. C2, Campo Grande, 1749- 016 Lisbon Portugal; 2 Universidade de Lisboa, Lisbon, Portugal Universidade de Lisboa Lisbon Portugal; 3 Centre for Ecology, Evolution and Environmental Changes (cE3c)/Azorean Biodiversity Group, CHANGE – Global Change and Sustainability Institute, Faculty of Agricultural Sciences and Environment, University of the Azores, Rua Capitão João d´Ávila, Pico da Urze, Angra do Heroísmo, Azores, Portugal Centre for Ecology, Evolution and Environmental Changes (cE3c)/Azorean Biodiversity Group, CHANGE – Global Change and Sustainability Institute, Faculty of Agricultural Sciences and Environment, University of the Azores, Rua Capitão João d´Ávila, Pico da Urze Angra do Heroísmo, Azores Portugal; 4 Centre for Functional Ecology, Department of Life Sciences, University of Coimbra, Calçada Martim de Freitas, 3000-456, Coimbra, Portugal Centre for Functional Ecology, Department of Life Sciences, University of Coimbra, Calçada Martim de Freitas, 3000-456 Coimbra Portugal; 5 Departamento de Ecologia y Biologia Animal, Universidad de Vigo, Vigo, Spain Departamento de Ecologia y Biologia Animal, Universidad de Vigo Vigo Spain; 6 IUCN SSC Mid-Atlantic Islands Specialist Group, Angra do Heroísmo, Azores, Portugal IUCN SSC Mid-Atlantic Islands Specialist Group Angra do Heroísmo, Azores Portugal

**Keywords:** biodiversity conservation, diversity assessment, habitat associations, miombo forest, Mozambique, new records

## Abstract

**Background:**

The Gorongosa National Park (Mozambique) is one of the most emblematic protected areas in Africa, well known for its vertebrate biodiversity and restoration ecology efforts following the Mozambican civil war in 1992. The invertebrate biodiversity of Gorongosa National Park is still poorly studied, although the scarce information available indicates the existence of a rich number of species, namely in the case of tiger- and ground-beetles (Coleoptera, Caraboidea). Moreover, the study of arthropod assemblages is key for designing conservation practices since they are potentially accurate biodiversity and ecological indicators. Hence, the diversity assessment of Caraboidea beetles using standardised methodologies is likely to provide a new insight for future conservation planning and help to quantify the effects of climate change in areas identified as vulnerable to anthropogenic pressures, such as the Gorongosa National Park.

**New information:**

We report the occurrence of five tiger beetles (Cicindelidae) and 93 ground-beetles (Carabidae) species/morphospecies in Gorongosa National Park from a field survey funded by the ECOASSESS project. Sampling was performed in the four main habitat types present in the Park (miombo tropical forest, mixed dry forest, transitional forest and grasslands) between 25 October and 25 November 2019. In this sampling window, the turnover of Caraboidea species from the dry season to the wet season was recorded for the first time. Twenty-eight species of ground-beetles are new records to Mozambique, including three new subgenera and three new genera. Additional information on species phenology and habitat preferences is also provided.

## Introduction

Mozambique is a large southern African country covered mostly by a miombo-type of savannah, dominated by Caesalpinioideae woodlands (Malmer 2007), while true forests comprise a minor area, such as the rain forests on the slopes of Mount Gorongosa (e.g. White (1983)). The major threats to Mozambican ecosystems and biodiversity include, amongst others, natural resources overexploitation, habitat fragmentation, fires and pollution (Timberlake 2000). Yet, since the end of the Mozambican civil war in 1992 - and particularly after 2005 - the Gorongosa National Park (GNP) has become a key protected area for biodiversity conservation and wildlife restoration with special focus on emblematic megafauna (Dunham 2004, Stalmans 2012, Bouley et al. 2018, Branco 2018, Bouley et al. 2021). GNP comprises a heterogeneous landscape with four main habitats in the low plateau of the Park, namely miombo tropical forest, mixed dry forest, grassland and transitional forest (Stalmans et al. 2019). These habitat types are subjected to marked seasonal changes due to the annual flooding of Lake Urema in the wet season. This contrasting seasonality greatly influences the GNP landscape and dynamics of wildlife (Bohme 2005, Beilfuss et al. 2007), particularly the biodiversity of soil fauna.

Several environmental and human-related pressures are potential threats to soil fauna communities of Gorongosa. Flooding dynamics and landscape configuration in GNP could experience dramatic alterations due to the effects of climate change. An increase in the intensity and duration of the dry season, as well as more frequent extreme events (e.g. heat waves and heavy rainfalls) have been observed recently and are expected to increase in the next decades (Hulme et al. 2001, Beilfuss et al. 2007, Tadross 2009, Niang et al. 2014, Jinga 2019, Engdaw et al. 2022). A decrease in vegetation cover is occurring throughout the Sofala Province where the Park is situated (World Food Programme 2018) and two of the three most common trees in the GNP are highly susceptible to longer dry periods (Massad and Castigo 2016). Human presence around the Park is also a driving pressure. Agricultural and deforestation practices on the Gorongosa Mountain contribute to the deterioration of the hydrological system that feeds the GNP (Beilfuss et al. 2007, Walker 2015). Soil fauna and, particularly, Caraboidea beetles, will be strongly influenced by direct and indirect effects of climatic changes such as alterations in habitat structure and composition and in abiotic conditions, like air temperature, soil moisture and erosion events (Brandmayr and Pizzolotto 2016, Knisley et al. 2016, Jaskuła et al. 2019, Kirichenko-Babko et al. 2020, Avtaeva et al. 2021). Therefore, monitoring studies in climatically vulnerable areas are determinants to evaluate the effects of future climate change on Caraboidea diversity and community composition in GNP.

Caraboidea beetles encompass more than 40,000 known species worldwide (Desender et al. 1994, Lövei and Sunderland 1996, Lorenz 2019). Tiger- and ground-beetles can have a wide variety of ecological roles and feeding habits (Kotze et al. 2011), comprising carnivorous, herbivorous and omnivorous species, i.e. occupying a large range of trophic levels (Johnson and Cameron 1969,Honek et al. 2003, Riddick 2008). Consequently, they have been used as model organisms and as ecological and biodiversity bioindicators in rapid assessments and monitoring studies in the Nearctic and Palearctic Regions (Desender et al. 1994, Pearson and Cassola 2007, Work et al. 2008, Lemić et al. 2017, Mazzei et al. 2017, Cherine et al. 2019). Yet, only a few studies have addressed standardised biodiversity studies focusing on Caraboidea communities, in tropical ecosystems from the southern African region (e.g. Samways et al. (1996), Lawes et al. (2005)). The entomofauna of Mozambique, including the Caraboidea, has been studied since the middle of the 19^th^ century and most of the insect specimens were collected under zoological/entomological expeditions carried out by institutions or by individual persons (e.g. travellers, missionaries, naturalists). Caraboidea material collected is, therefore, scattered and usually reported as new records or new taxa in several publications and monographic works (e.g. Klug (1853), Péringuey (1896), Basilewsky(a) (1950), Basilewsky(b) (1950), Basilewsky (1951), Straneo (1958), Basilewsky (1963), Lecordier(a) (1978), Lecordier(b) (1978), Schüle (2004), Cassola and Bouyer (2007), Schüle (2011), Kleinfeld and Puchner (2012), Serrano (2014)), but never in consistent and systematic focused works. In this pioneering study, we aimed to increase the knowledge on Caraboidea beetle diversity in the four main habitats of the GNP. The results will provide the baseline data that could improve future monitoring programmes on Caraboidea diversity and community changes, leading to a better design of conservation strategies and evaluating the impacts of climate change on GNP.

## General description

### Purpose

Our main goal was to assess the soil fauna diversity in the main habitat types of the low plateau of Gorongosa National Park (GNP). Several invertebrate assemblages were surveyed, concretely as Annelida, Collembola, Formicidae, Tenebrionidae, Scarabaeoidea and Caraboidea (Coleoptera, Cicindelidae, Carabidae). The final aim was to increase the knowledge on the Caraboidea fauna associated with different habitat types, building a baseline to support further studies on tiger- and ground-beetle diversity trends and community changes in future monitoring programmes (e.g. to assess the effects of climate change and other anthropogenic disturbances).

## Project description

### Title

Caraboidea from Gorongosa National Park

### Study area description

**Figure 1.**
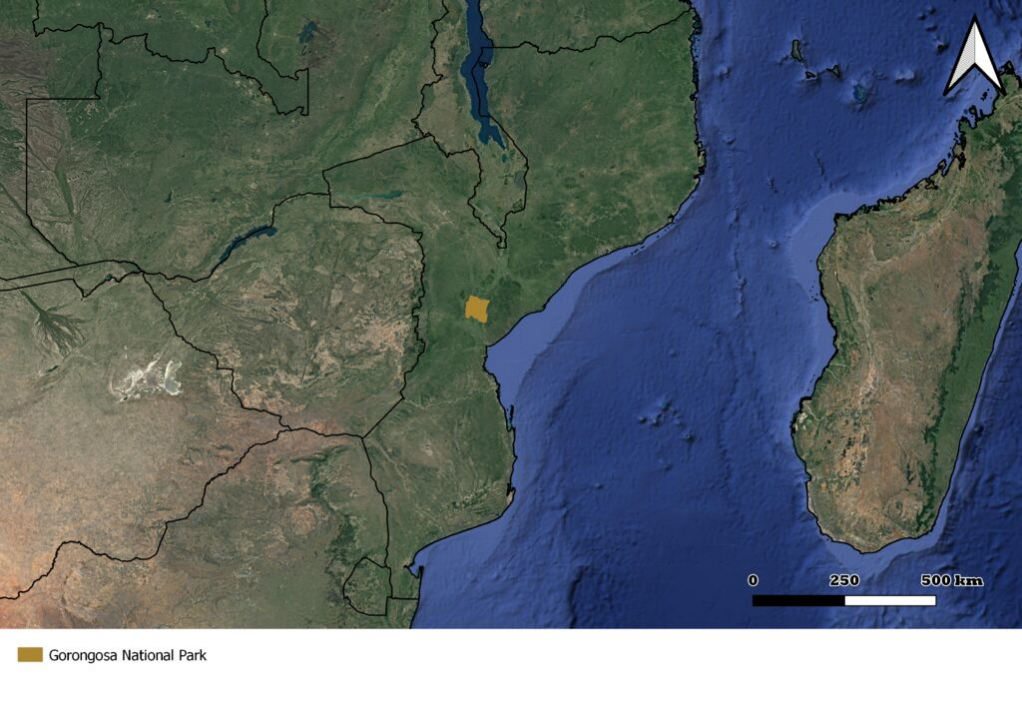
Location of the Gorongosa National Park in Mozambique.

Fieldwork was carried out in the main habitat types covering the low plateau of the GNP, namely the miombo forest, mixed dry forest, transitional forest and grasslands (Stalmans and Beilfuss 2008). GNP is located in the centre of Mozambique, occupying around 4000 km^2^ of the Sofala Province (Stalmans et al. 2019) (Fig. 1) with altitudes ranging from 15-80 m in the valley to 300-400 m above sea level in the hills surrounding the basin (Stalmans et al. 2019). This region has a tropical climate with mean annual precipitation of 700-900 mm, along with two distinct seasons (dry and wet). Between 2000 and 2016, a decrease in precipitation was recorded in Gorongosa (Herrero et al. 2020). GNP annual temperatures range between 15º and 30ºC, with warmer temperatures usually recorded in the wet season (Herrero et al. 2020). This rainy season occurs in the months of November to April and is associated with heavy rainfall, resulting in extensive flooding around Lake Urema, located in the centre of the low plateau. In this low plateau of the Park (“lower Gorongosa”), the dominant habitat types range from open savannahs (grasslands) to mixed savannahs (transitional forests) and forested habitat types comprising mixed forests and miombo forests. The latter is dominated by trees of the genus *Brachystegia* (Herrero et al. 2020).

### Funding

This study was supported by the Project ECOASSESS – A biodiveristy and ECOlogical ASSESSment of soil fauna of Gorongosa National Park (Mozambique) (PTDC/BIA¬CBI/29672/2017) funded through national funds by FCT / MCTES (PIDDAC) under the Programme All Scientific Domains. Marie Bartz was contracted by the University of Coimbra (contract nr. IT057-19-7955) through financial support by the Project/R&D Instituition ECOASSESS. Sara Mendes was financially supported by FCiências – Associação para a investigação e Desenvolvimento de Ciências through research grants funded by the Project/R&D Institution ECOASSESS. Mário Boieiro and Sérgio Timóteo were supported by FCT under contracts DL57/2016/CP1375/CT0001 and CEECIND/00135/2017, respectively. ECOASSESS field sampling was carried out with the logistic support of Gorongosa National Park under supervision of Jason Denlinger (Lab manager) and Mark Stalmans (Director of Scientific Service).

## Sampling methods

### Study extent

ECOASSESS survey focused on the four main habitat types, i.e. miombo tropical forest, mixed dry forest, transition forest and grasslands (Fig. 2), encompassing the low plateau of the Gorongosa National Park, in a total sampling area of 56,130 m^2^. These habitats were selected considering the ecosystem changes and complex dynamics due to seasonal flooding and human disturbance in this area of the Park. Within each habitat type, 25 sampling plots were randomly distributed (Fig. 3), with a minimum distance of 1 km between each other (Table 1).

### Sampling description

Caraboidea beetle sampling was done through the use of pitfall traps (Drift 1951, Greenslade 1964). In each sampling plot, three pitfall traps were arranged in the shape of a triangle with 5 m of separation between them. Pitfall traps consisted of plastic cups with 10 cm diameter and filled with ethyleneglycol (5%). To include data from the transition between the dry and wet seasons, Caraboidea beetles were collected during three sampling periods: T1 (25 October to 5 November of 2019) and T2 (5-15 November of 2019), both during the dry season and T3 (15-25 November of 2019) in the wet season, comprising ten days per sampling window. During pitfall sampling, the content of each pitfall was enclosed in a cloth bag and all bags were put together in jerricans filled with 96% ethanol. Afterwards, all jerricans were transported to the laboratory at the Centre for Ecology, Evolution and Environmental Changes (University of Lisbon, Portugal) for sorting and taxonomic identification of Caraboidea beetle specimens. All other taxa were separated and stored in 75% ethanol for further possible studies. Taxonomic identification was performed to the species/subspecies level or morphospecies. Data from pitfall sub-samples were then pooled before data analyses.

### Quality control

All carabid and cicindelid specimens were taxonomically identified by Artur R. M. Serrano. Whenever possible, the identification was made to the subspecies or species level, otherwise, the specimens were separated as morphospecies.

## Geographic coverage

### Description

Gorongosa National Park, Gorongosa, Sofala, Mozambique

### Coordinates

-19.05286 and -18.86422 Latitude; 34.15946 and 34.49303 Longitude.

## Taxonomic coverage

### Taxa included

**Table:** 

Rank	Scientific Name	Common Name
family	Carabidae	Ground Beetles
family	Cicindelidae	Tiger Beetles

## Temporal coverage

**Data range:** 2019-10-25 – 2019-11-25.

## Collection data

### Specimen preservation method

All separated specimens were preserved in 75% ethanol.

## Usage licence

### Usage licence

Creative Commons Public Domain Waiver (CC-Zero)

## Data resources

### Data package title

Inventory of tiger- and ground-beetles (Coleoptera Caraboidea, Cicindelidae, Carabidae) from the Gorongosa National Park (Mozambique)

### Resource link

http://ipt.gbif.pt/ipt/resource?r=goundbeetles_mozambique

### Alternative identifiers

https://www.gbif.org/dataset/ced770f9-7dd5-49c6-8030-795dd409921a

### Number of data sets

1

### Data set 1.

#### Data set name

Inventory of tiger- and ground-beetles (Coleoptera Caraboidea, Cicindelidae, Carabidae) from the Gorongosa National Park (Mozambique)

#### Data format

Darwin Core Archive format

#### Character set

UTF-8

#### Download URL

http://ipt.gbif.pt/ipt/archive.do?r=goundbeetles_mozambique

#### Data format version

Version 1.8

#### Description

Our project reported the occurrence of five tiger-beetles (Cicindelidae) and 93 species/morphospecies of ground-beetles (Carabidae) in Gorongosa National Park, ascertained through a field survey supported by the ECOASSESS project. The sampling activities were conducted between 25 October and 25 November, encompassing the Park's four principal habitat types, namely miombo tropical forest, mixed dry forest, transitional forest and grasslands. This survey period allowed us to document, for the first time, the changes in Caraboidea species diversity from the dry season to the wet season. Amongst the noteworthy records are 28 ground-beetle species that represent new records for Mozambique, including three novel subgenera and three previously unrecorded genera. Furthermore, we offer supplementary insights into species phenology and habitat preferences.

The dataset submitted to GBIF is structured as a sample event dataset, with two tables: event (as core) and occurrences. The data in this sampling event resource have been published as a Darwin Core Archive (DwC-A), which is a standardised format for sharing biodiversity data as a set of one or more data tables. The core data tables contain 403 event and 838 occurrence records (Serrano et al. 2023).

**Data set 1.** 

Column label	Column description
Table of Sampling Events	Table with sampling events data (beginning of table).
id	Unique identification code for sampling event data.
eventID	Identifier of the events, unique for the dataset.
samplingProtocol	The sampling protocol used to capture the species.
sampleSizeValue	The volume of liquid used for each sample.
sampleSizeUnit	The unit of the sample size value.
samplingEffort	The amount of time of each sampling.
eventDate	Date range when the record was collected.
habitat	The surveyed habitat.
country	Country of the sampling site.
country code	ISO code of the country of the sampling site.
municipality	Municipality of the sampling site.
locality	Locality of the sampling site.
verbatimElevation	The original description of elevation (altitude, usually above sea level), in metres.
eventRemarks	A reference to the protocol used to determine the measurement (measurement method).
decimalLatitude	Approximate centre point decimal latitude of the field site in GPS coordinates.
decimalLongitude	Approximate centre point decimal longitude of the field site in GPS coordinates.
geodeticDatum	The ellipsoid, geodetic datum or spatial reference system (SRS) upon which the geographic coordinates given in decimalLatitude and decimalLongitude are based.
coordinateUncertaintyInMetres	Uncertainty of the coordinates of the centre of the sampling plot.
coordinatePrecision	Precision of the coordinates.
georeferenceSources	A list (concatenated and separated) of maps, gazetteers or other resources used to georeference the Location, described specifically enough to allow anyone in the future to use the same resources.
Table of Species Occurrence	Table with species abundance data (beginning of new table).
id	Unique identification code for species abundance data.
type	Type of the record, as defined by the Public Core standard.
licence	Reference to the licence under which the record is published.
institutionID	The identity of the institution publishing the data.
collectionID	The identity of the collection publishing the data.
institutionCode	The code of the institution publishing the data.
collectionCode	The code of the collection where the specimens are conserved.
datasetName	Name of the dataset.
basisOfRecord	The nature of the data record.
dynamicProperties	The name of the scientific project funding the sampling.
occurrenceID	Identifier of the record, coded as a global unique identifier.
recordedBy	Name of the person who performed the sampling of the specimens.
organismQuantity	Total number of individuals captured.
sex	The sex and quantity of the individuals captured.
organismQuantityType	Informs about the type of the entity that is quantified.
identifiedBy	Name of the person who identified the specimens.
dateIdentified	Date when the specimens were identified.
identificationRemarks	Description of the observed wing traits.
scientificName	Complete scientific name including author and year.
kingdom	Kingdom name.
phylum	Phylum name.
class	Class name.
order	Order name.
family	Family name.
genus	Genus name.
subgenus	Subgenus name.
specificEpithet	Specific epithet.
infraspecificEpithet	Infraspecific Epithet.
taxonRank	Lowest taxonomic rank of the record.
scientificNameAuthorship	Name of the author of the lowest taxon rank included in the record.
taxonRemarks	Scientific name with mention of cases of subgenera with stautus "subg. incertae" and "s. str.".

## Additional information

### Results

A total of 1777 Caraboidea beetle specimens were identified, of which 1765 were identified to species or subspecies. They were from 98 different species/morphospecies (5 Cicindelidae and 93 Carabidae) (Table 2, Serrano et al. (2023)). Only 785 out of the 900 pitfalls were collected (Table 3), either due to trap destruction or plot inaccessibility in the wet season due to flooding. Considering the last checklist including information on Mozambique Caraboidea (Lorenz 2019), there are three genera (*Apristus* Chaudoir, 1846; *Platytarus* Fairmaire, 1850; *Crepidogastrillus* Basilewsky, 1959), three subgenera (*Klugipaussus* Kolbe, 1927; *Tyronia* Liebke, 1934; *Trechicus* LeConte, 1853) and 28 species/subspecies that are new records for this country (Table 2). Additionally,, most of the species/subspecies sampled in this study had never been recorded for GNP and of the few that were, it was only for the Chitengo area (e.g. Alves (1974), Schüle (2011)).

Licininae and Lebiinae were the two Caraboidea subfamilies recording the highest number of species (15 species each), while the most abundant specimens belonged to the subfamily Brachininae (third most speciose with 12 species). The most abundant genera were *Pheropsophus* Solier, 1833 (Brachininae), *Microlestes* Schmidt-Goebel, 1846 (Lebiinae), *Chlaenius* Bonelli, 1810 (Licininae) and *Abacetus* Dejean, 1828 (Pterostichinae) (Table 2). At the species level, *Microlesteszambezianus* (Mateu, 1960) (Lebiinae) and *Pheropsophusmashunus* (Péringuey, 1896) (Brachininae) were the most abundant, while *Chlaeniusconformis* (Dejean, 1831), *Phesopsorusinsignisinsignis* (Boheman, 1848) and *Graphipterustristis* (Klug, 1853) were the most well-represented, i.e. the only ones present across all habitat types (Table 2).

A considerable number of caraboid species were recorded only once (39 singletons, comprising 39.8% of the total assemblage) or twice (6 doubletons, comprising 6.1% of the total assemblage), indicating that almost 50% of the Caraboidea sampled in the GNP are rare species. The presence of rare species (singletons and doubletons) was common across all habitat types, but their number was highest in the mixed and transitional forests (Table 2). On the other hand, we found that two to five species were generally dominant in the Caraboidea assemblages, but species identity varied amongst habitat types (Table 2).

Transitional forest recorded the highest number in Caraboidea specimens (Table 3), with the dominance of *P.insignisinsignis*, *P.mashunus*, *Distichuspicicornis* (Dejean. 1831), *Tetragonoderusimmaculatus* LaFerté-Sénectère, 1853, *Microlestesflavipesmicromys* Alluaud, 1918 and *M.zambezianus*. Grassland recorded the second highest amount of Caraboidea specimens, with *D.picicornis*, *Abacetus perturbator Péringuey*, 1899, *Chlaeniusdiscopictusnuncius* Péringuey, 1908 and also *M.zambezianus* as the most abundant species. Mixed dry forest was the third habitat type in terms of number of specimens of Caraboidea collected in pitfalls, with the dominance of *Crepidogasterlangenhani*, *Scaritestenebricosusmolossus* Klug, 1853, *Abacetuspercoides* Fairmaire, 1868 and *Orthotrichusinsolitum* (Péringuey 1896). Miombo forest recorded the lowest number of Caraboidea specimens (Table 3) and *Crepidogasterlangenhani* Liebke, 1927 as well as *P.mashunus* were the dominant species in this habitat type.

Amongst the 98 species/subspecies recorded in this study, only a total of 24 were found across the three sampling seasons. The wet season recorded the highest absolute values in species numbers across habitats, but the abundance values in pitfalls varied according to the habitat type (Table 3). Only miombo and mixed dry forests recorded a similar pattern between abundance and species numbers found in the pitfall traps.

Our results contribute to fill the gap in the description of Caraboidea communities across the main habitat types of the GNP, setting the stage for the creation of baseline data for future assessments and comparisons with other studies. Our survey also provides a reference values for individual species that could support conservation schemes aiming to evaluate the effects of climate change on richness and diversity patterns of Caraboidea beetles in GNP.

## Figures and Tables

**Figure 2a.**
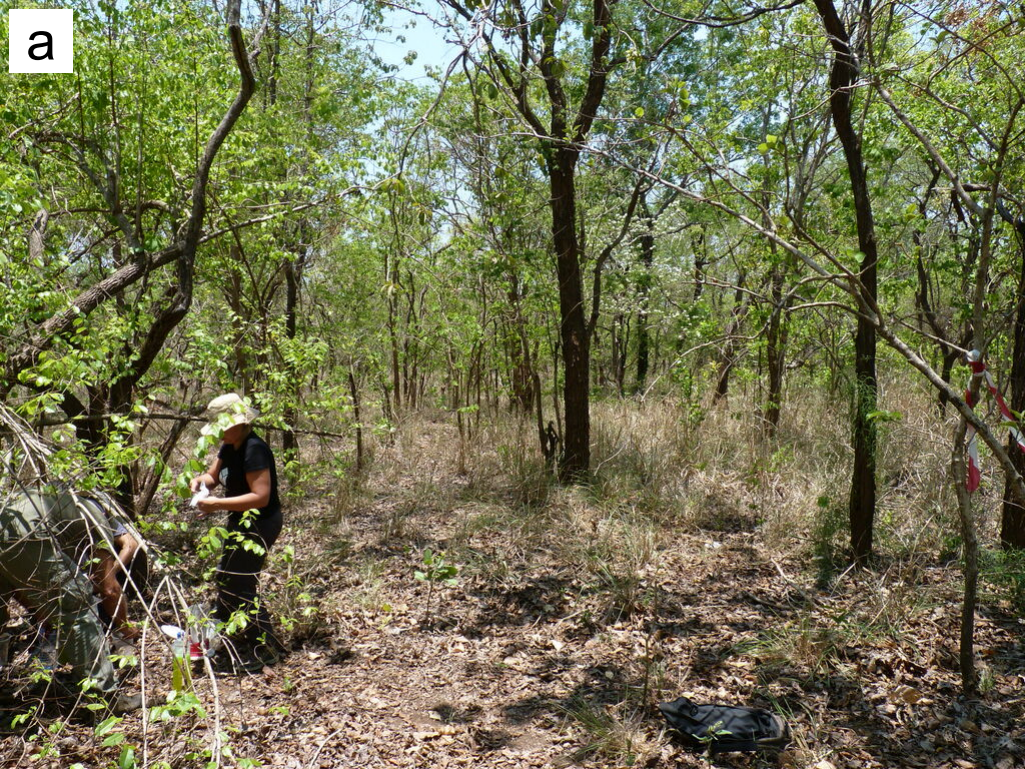
Miombo tropical forest.

**Figure 2b.**
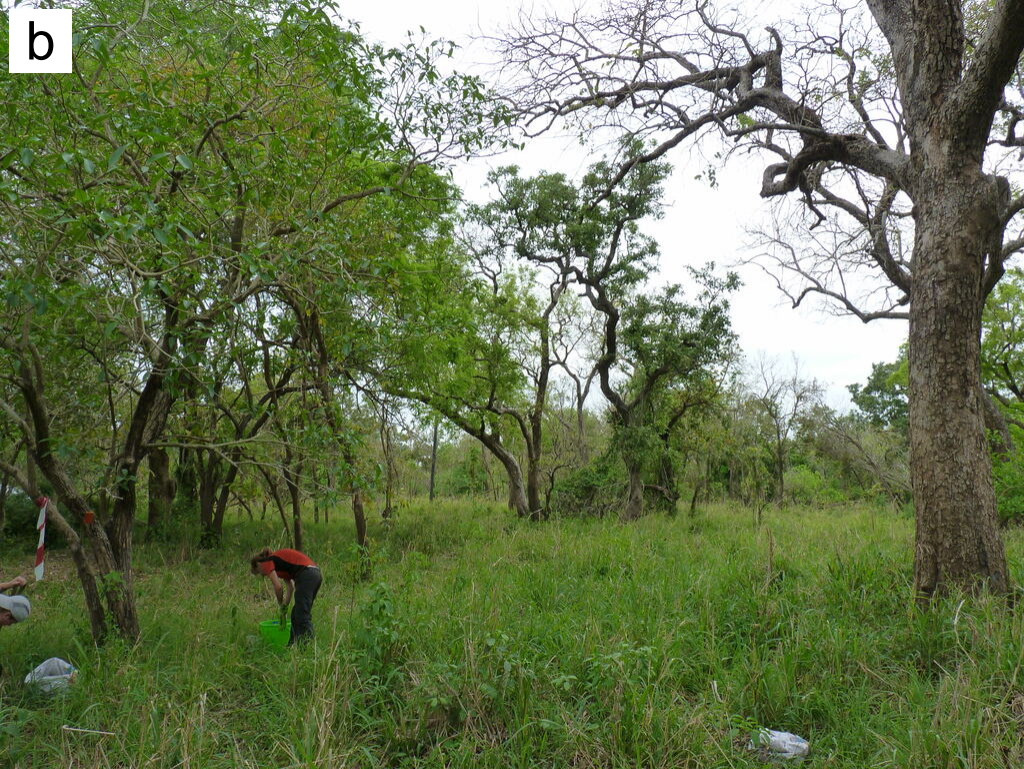
Mixed dry forest.

**Figure 2c.**
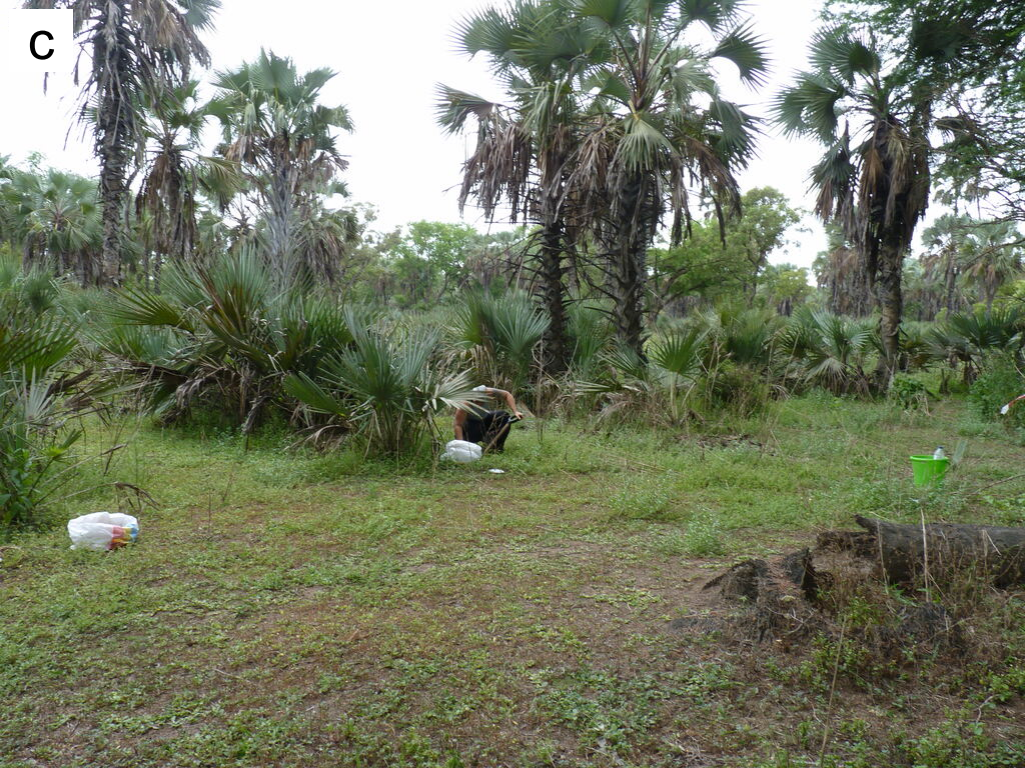
Transition forest.

**Figure 2d.**
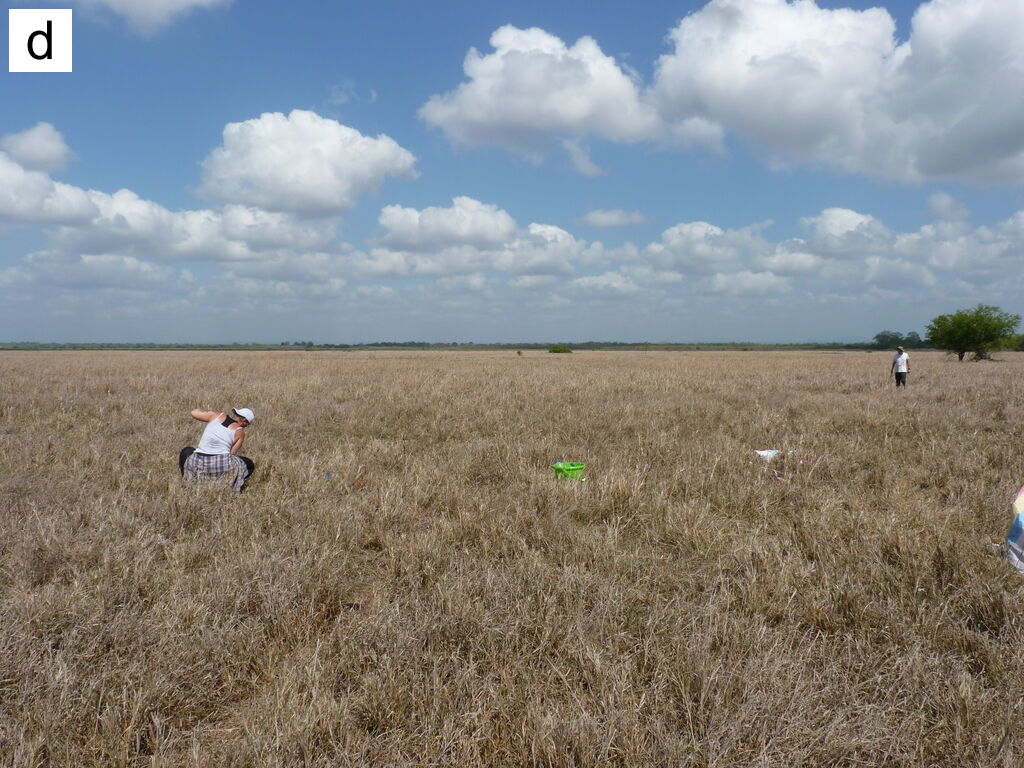
Grassland.

**Figure 3a.**
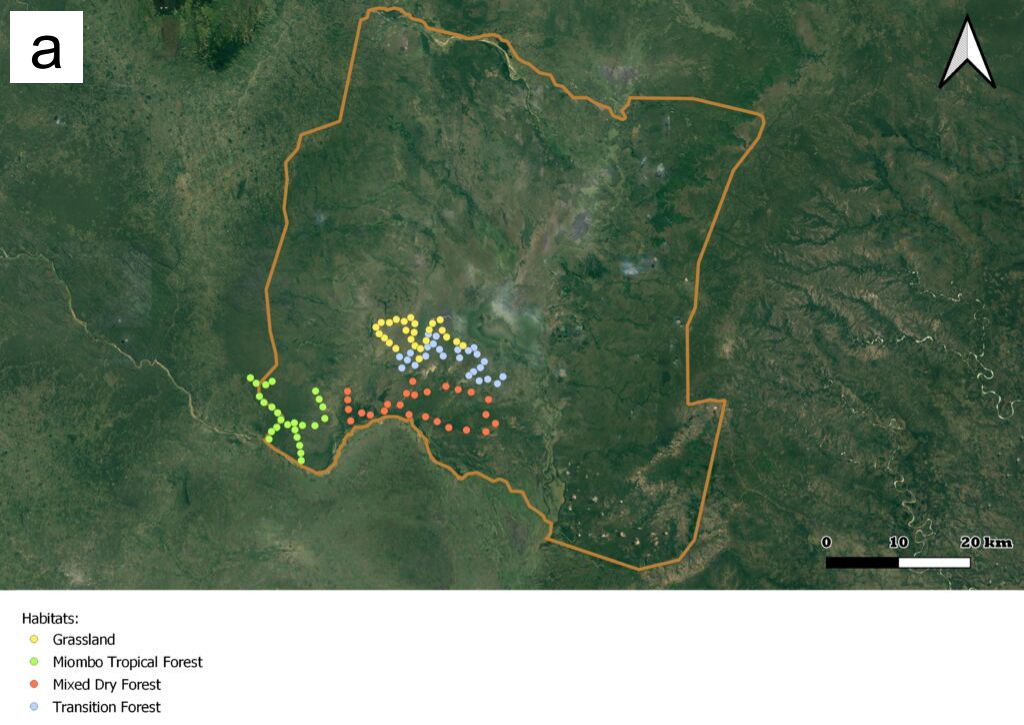
Location of sampling plots within the GNP;

**Figure 3b.**
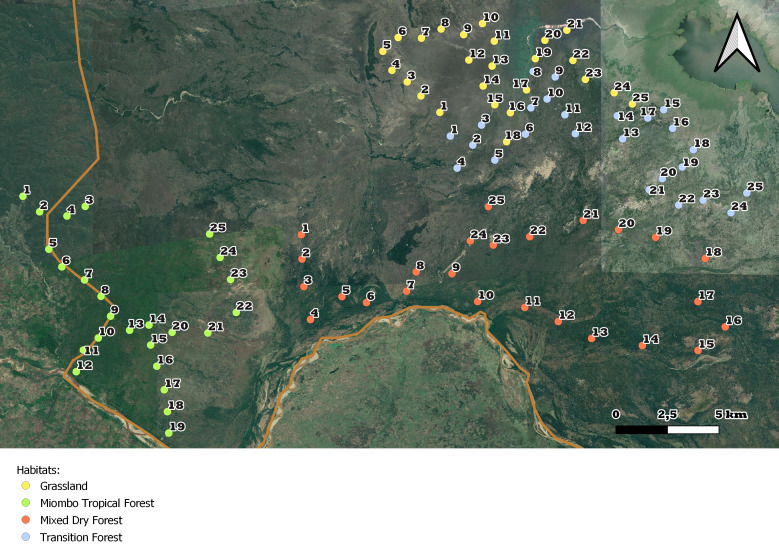
Close-up of the distribution of the sampling plots per habitat type.

**Table 1.** Geographic coordinates of the sampling plots in the four main habitat types.

Plot	Longitude	Latitude
Mixed Dry Forest 1	34.28777	-18.96146
Mixed Dry Forest 2	34.28807	-18.97271
Mixed Dry Forest 3	34.28896	-18.98538
Mixed Dry Forest 4	34.29197	-19.00057
Mixed Dry Forest 5	34.30649	-18.99001
Mixed Dry Forest 6	34.31777	-18.99275
Mixed Dry Forest 7	34.33626	-18.98747
Mixed Dry Forest 8	34.34068	-18.97864
Mixed Dry Forest 9	34.35718	-18.97943
Mixed Dry Forest 10	34.36901	-18.99226
Mixed Dry Forest 11	34.39071	-18.99499
Mixed Dry Forest 12	34.40616	-19.00148
Mixed Dry Forest 13	34.42152	-19.00931
Mixed Dry Forest 14	34.44492	-19.01258
Mixed Dry Forest 15	34.47054	-19.01483
Mixed Dry Forest 16	34.48309	-19.00394
Mixed Dry Forest 17	34.47051	-18.99229
Mixed Dry Forest 18	34.47388	-18.97243
Mixed Dry Forest 19	34.45102	-18.96265
Mixed Dry Forest 20	34.43388	-18.95914
Mixed Dry Forest 21	34.41764	-18.95491
Mixed Dry Forest 22	34.39302	-18.96239
Mixed Dry Forest 23	34.37619	-18.96627
Mixed Dry Forest 24	34.36562	-18.96432
Mixed Dry Forest 25	34.37392	-18.94854
Grassland 1	34.35158	-18.90512
Grassland 2	34.34286	-18.89755
Grassland 3	34.33655	-18.89112
Grassland 4	34.32949	-18.88578
Grassland 5	34.32532	-18.87699
Grassland 6	34.33233	-18.87067
Grassland 7	34.34311	-18.87095
Grassland 8	34.35215	-18.86675
Grassland 9	34.36256	-18.86932
Grassland 10	34.37122	-18.86422
Grassland 11	34.37667	-18.87231
Grassland 12	34.36494	-18.88102
Grassland 13	34.37567	-18.8838
Grassland 14	34.37153	-18.893
Grassland 15	34.37691	-18.90161
Grassland 16	34.38407	-18.90527
Grassland 17	34.39153	-18.89477
Grassland 18	34.38234	-18.91865
Grassland 19	34.39555	-18.88038
Grassland 20	34.4	-18.87191
Grassland 21	34.41009	-18.86726
Grassland 22	34.41291	-18.88118
Grassland 23	34.41865	-18.8899
Grassland 24	34.43191	-18.8961
Grassland 25	34.44029	-18.90127
Miombo Tropical Forest 1	34.15946	-18.9438
Miombo Tropical Forest 2	34.16716	-18.95094
Miombo Tropical Forest 3	34.18818	-18.94843
Miombo Tropical Forest 4	34.17975	-18.95287
Miombo Tropical Forest 5	34.1714	-18.96817
Miombo Tropical Forest 6	34.17742	-18.9763
Miombo Tropical Forest 7	34.18785	-18.98234
Miombo Tropical Forest 8	34.19546	-18.98988
Miombo Tropical Forest 9	34.19985	-18.99903
Miombo Tropical Forest 10	34.19418	-19.00907
Miombo Tropical Forest 11	34.18733	-19.01463
Miombo Tropical Forest 12	34.18403	-19.02461
Miombo Tropical Forest 13	34.20862	-19.00551
Miombo Tropical Forest 14	34.21755	-19.00312
Miombo Tropical Forest 15	34.2183	-19.01233
Miombo Tropical Forest 16	34.22114	-19.02208
Miombo Tropical Forest 17	34.22458	-19.03293
Miombo Tropical Forest 18	34.22604	-19.043
Miombo Tropical Forest 19	34.22668	-19.05286
Miombo Tropical Forest 20	34.2282	-19.00645
Miombo Tropical Forest 21	34.24467	-19.00678
Miombo Tropical Forest 22	34.25776	-18.99729
Miombo Tropical Forest 23	34.25516	-18.98212
Miombo Tropical Forest 24	34.25033	-18.97195
Miombo Tropical Forest 25	34.2455	-18.96117
Transition Forest 1	34.35642	-18.91604
Transition Forest 2	34.36676	-18.9202
Transition Forest 3	34.37078	-18.91097
Transition Forest 4	34.35954	-18.9308
Transition Forest 5	34.3769	-18.92711
Transition Forest 6	34.39099	-18.91516
Transition Forest 7	34.39353	-18.90303
Transition Forest 8	34.39458	-18.88629
Transition Forest 9	34.40474	-18.8888
Transition Forest 10	34.40099	-18.89897
Transition Forest 11	34.40921	-18.90624
Transition Forest 12	34.41402	-18.91494
Transition Forest 13	34.43582	-18.91736
Transition Forest 14	34.4333	-18.9067
Transition Forest 15	34.45476	-18.90391
Transition Forest 16	34.45885	-18.91251
Transition Forest 17	34.44741	-18.90774
Transition Forest 18	34.46841	-18.92232
Transition Forest 19	34.46325	-18.93033
Transition Forest 20	34.45408	-18.93565
Transition Forest 21	34.44806	-18.94075
Transition Forest 22	34.46164	-18.94781
Transition Forest 23	34.47288	-18.94556
Transition Forest 24	34.48573	-18.95137
Transition Forest 25	34.49303	-18.94227

**Table 2.** List of Caraboidea species and subspecies and their abundance in the different habitat types during the three sampling periods (T1: 25 October-5 November; T2: 5-15 November; T3: 15-25 November 2019). New records at the Species, Subgenus or Genus levels are also provided (Sp, SbG and G, respectively). The first five species belong to the family Cicindelidae and so they are not included in any subfamily.

Species	Subfamily	New Record for Mozambique	Miombo Tropical Forest			Mixed Dry Forest			Transitional Forest			Grassland			Total
			T1	T2	T3	T1	T2	T3	T1	T2	T3	T1	T2	T3	
*Manticorascabra* Klug, 1849	NA		0	0	7	0	0	0	0	0	0	0	0	0	7
*Megacephalaasperata* (Waterhouse, 1877)	NA		0	0	11	0	2	10	0	0	0	0	0	0	23
*Dromicadolosalatepolita* Schüle, 2011	NA		0	0	0	0	0	4	0	0	0	0	0	0	4
*Prothymidiaangusticollis* (Boheman, 1848)	NA		0	0	0	0	0	4	0	0	0	0	0	0	4
*Ellipticacompressicorniscompressicornis* (Boheman, 1861)	NA		0	0	1	0	0	0	0	0	0	0	0	0	1
*Pentaplatarthrusgestroi* Kolbe, 1896	Paussinae		0	0	1	0	0	0	0	0	0	0	0	0	1
Paussus (Bathypaussus) cultratus Westwood, 1850	Paussinae	SbG	0	0	1	0	0	0	0	0	0	0	0	0	1
Paussus (Klugipaussus) pseudoklugi Luna de Carvalho, 1963	Paussinae		0	0	0	0	0	0	0	1	0	0	0	0	1
Crepidogaster(s. str.)langenhani (Liebke, 1927)	Brachininae	Sp	0	0	18	4	5	17	2	0	0	0	0	0	46
Crepidogaster(s. str.)protuberata Basilewsky, 1959	Brachininae		1	1	3	0	0	0	0	0	0	0	0	0	5
Crepidogaster (Tyronia) longelineata (Basilewsky, 1988)	Brachininae	SbG	0	1	3	1	0	4	0	0	0	0	0	0	9
*Crepidogastrilluscurtulus* Basilewsky, 1959	Brachininae	G	0	0	0	0	1	0	0	0	0	0	0	0	1
Pheropsophus (Stenaptinus) dregei Chaudoir, 1876	Brachininae		0	0	0	0	0	0	0	0	4	4	0	6	14
Pheropsophus (Stenaptinus) insignis insignis (Boheman, 1848)	Brachininae		0	0	1	0	0	14	23	21	56	2	4	3	124
Pheropsophus (Stenaptinus) mashunus Péringuey, 1896	Brachininae		6	15	14	0	0	11	160	114	46	0	0	0	366
Pheropsophus (Stenaptinus) stenopterus Chaudoir, 1878	Brachininae		0	0	0	0	0	0	0	0	3	2	0	0	5
Styphlomerus(s. str.)neaveineavei Liebke, 1934	Brachininae	Sp	2	1	0	4	2	3	1	1	1	0	0	0	15
Brachinus (subg. incertae) distans Lorenz, 1998	Brachininae		0	0	0	0	0	0	0	0	0	1	0	4	5
Brachinus (subg. incertae) laetus Dejean, 1831	Brachininae	Sp	0	0	0	0	0	0	0	0	0	1	0	1	2
Brachinus (subg. incertae) leprieuri Gory, 1833	Brachininae	Sp	0	0	0	0	0	0	0	0	0	0	0	1	1
Calosoma (Ctenosta) planicolle Chaudoir, 1869	Carabinae		0	0	0	0	0	1	0	0	0	0	0	0	1
*Siagonacaffra* Boheman, 1848	Siagoninae		0	0	0	0	0	0	0	0	1	0	1	0	2
*Siagonalevasseuri* Lecordier, 1970	Siagoninae		0	0	0	0	0	0	0	0	1	0	0	0	1
*Siagonapartita* Lecordier, 1979	Siagoninae		0	0	0	0	0	0	0	0	1	0	0	0	1
Distichus(s. str.)bisquadripunctatus (Klug, 1862)	Scaritinae		0	0	0	0	0	0	1	0	1	0	0	1	3
Distichus(s. str.)picicornis (Dejean, 1831)	Scaritinae		0	0	0	0	0	0	12	5	3	1	3	18	42
*Scaritesaestuans* Klug, 1853	Scaritinae		0	0	0	0	0	0	0	0	0	1	2	8	11
Scarites(s. str.)tenebricosusmolossus Klug, 1853	Scaritinae		0	0	7	1	1	18	0	0	2	0	0	0	29
*Melaenuselegans* Dejean, 1831	Melaeninae		0	0	0	0	0	0	0	0	2	0	0	1	3
Cymbionotum(s. str.)schueppelii (Dejean, 1825)	Melaeninae		0	0	0	0	0	0	0	0	0	0	0	1	1
*Apotomusannulaticornis* Péringuey, 1896	Apotominae	Sp	0	0	0	0	0	0	0	0	1	0	0	0	1
*Apotomus* sp.2	Apotominae		0	0	0	0	0	0	0	0	0	0	0	1	1
Elaphropus(s. str.)aethiopicus Chaudoir, 1876	Trechinae		2	2	6	0	1	4	0	0	0	0	1	0	16
*Elaphropus* (s. str.) sp.	Trechinae		0	0	0	1	0	0	0	0	0	0	0	0	1
Elaphropus (Sphaeorotachys) haemorrhoidalis (Ponza, 1805)	Trechinae	Sp	0	0	0	0	2	0	0	0	0	0	0	0	2
Tachys (Paratachys) iridipennis Chaudoir, 1876	Trechinae	Sp	0	0	0	0	0	0	0	0	1	0	0	0	1
Tachys (Paratachys) sp.1	Trechinae		0	0	0	0	0	0	0	0	0	0	1	1	2
Tachys (Paratachys) sp.2	Trechinae		0	0	0	0	0	0	0	0	1	0	0	0	1
Abacetus (Distrigus) denticollis Chaudoir, 1878	Pterostichinae		0	0	3	0	0	0	0	0	0	0	0	0	3
Abacetus (Distrigus) nigrinus (Boheman, 1848)	Pterostichinae	Sp	0	0	0	0	0	0	0	0	3	0	0	1	4
Abacetus (Abacetus) percoides Fairmaire, 1868	Pterostichinae		1	1	8	1	3	55	0	0	0	0	0	0	69
Abacetus (Abacetus) pseudomashunus Straneo, 1950	Pterostichinae	Sp	0	0	0	0	0	0	0	0	1	0	0	0	1
Abacetus (Abacetus) sp.	Pterostichinae		0	0	0	0	0	0	0	0	1	0	0	0	1
Abacetus (Abacetillus) discolor (Roth, 1851)	Pterostichinae	Sp	0	0	0	1	0	11	0	0	0	0	0	0	12
Abacetus (Distrigodes) perturbator Péringuey, 1899	Pterostichinae	Sp	0	0	0	0	0	0	0	0	1	2	0	34	37
Abacetus (Astigis) cursor Péringuey, 1898	Pterostichinae	Sp	0	0	0	0	0	0	0	0	0	0	0	2	2
*Disphericus* sp.	Panagaeinae		0	0	0	0	0	1	0	0	0	0	0	0	1
*Tefflus carinatus carinatus* Klug, 1853	Panagaeinae		0	0	8	0	1	4	0	0	2	0	0	0	15
*Microschemus* sp.	Panagaeinae		1	0	0	0	0	0	0	0	0	0	0	0	1
*Systolocraniusgoryi* (Goryi, 1833)	Licininae		0	0	7	0	1	10	0	0	1	0	0	0	19
*Melanchitonlucidulus* (Boheman, 1848)	Licininae		0	0	0	0	0	0	0	0	0	0	0	1	1
Chlaenius (Pachydinodes) conformis Dejean, 1831	Licininae		1	2	0	1	0	0	0	0	1	1	0	2	8
Chlaenius (Prochlaeniellus) peringueyi Kuntzen, 1919	Licininae	Sp	0	0	0	0	0	0	1	0	5	0	0	6	12
Chlaenius (Pseudochlaeniellus) paenulatus Erichson, 1843	Licininae		0	0	0	0	0	0	0	0	0	0	0	1	1
Chlaenius (Chlaenionus) zanzibaricus giganteus (Péringuey, 1885)	Licininae		0	0	0	0	0	0	1	0	1	0	0	1	3
Chlaenius (Chlaeniostenus) cylindricollis Dejean, 1831	Licininae		0	0	0	0	0	0	9	5	11	3	1	6	35
Chlaenius (Amblygenius) sp.	Licininae		0	0	0	0	0	0	0	0	1	0	0	0	1
Chlaenius (Chlaenius) cosciniophorus Chaudoir, 1876	Licininae	Sp	0	0	0	0	0	0	0	2	0	0	0	1	3
Chlaenius (Chlaenius) discopictus nuncius Péringuey, 1908	Licininae	Sp	0	0	0	0	0	0	0	2	10	0	0	69	81
Chlaenius (Chlaenius) dusaultii diagraphus Alluaud, 1922	Licininae		0	0	0	0	0	0	0	0	1	0	0	0	1
Chlaenius (Chlaenius) notabilis La Ferté-Sénectère, 1851	Licininae		0	0	0	0	0	0	2	1	14	0	0	9	26
Chlaenius (Macrochlaenites) lugens Chaudoir, 1876	Licininae		0	0	0	0	0	0	1	0	1	0	0	4	6
Chlaenius (Paracallistoides) fulvicollis Chaudoir, 1876	Licininae		0	0	0	0	0	0	1	0	0	1	0	9	10
Chlaenius (Paracallistoides) kirki kirki Chaudoir, 1876	Licininae		0	0	0	0	0	3	0	0	0	0	0	0	3
*Notiobia* (*Diatypus)* sp.	Harpalinae		0	0	0	1	0	0	0	0	0	0	0	0	1
*Omostropusmandibularis* (Roth, 1851)	Harpalinae		0	0	0	0	0	1	0	0	0	0	0	0	1
*Parophonus* (*Hyparpalus) tomentosus* (Dejean, 1829)	Harpalinae		0	0	0	1	0	0	0	0	0	0	0	0	1
Siopelus (Haplocoleus) lucens Putzeys in Chaudoir, 1878	Harpalinae		0	0	0	0	0	0	0	0	1	0	0	0	1
Siopelus (Aulacoryssus) sp.	Harpalinae		0	0	0	0	0	1	0	0	0	0	0	0	1
*Orthotrichusinsolitum* (Péringuey, 1904)	Platyninae	Sp	0	0	0	0	2	47	0	0	0	0	0	0	49
Perigona (Trechicus) schmitzi (Basilewsky, 1989)	Lebiinae	SbG	0	0	0	0	1	0	0	0	0	0	0	0	1
*Graphipteruslineelus* Péringuey, 1896	Lebiinae		0	0	8	0	1	4	0	0	0	0	0	0	13
*Graphipterushornistaudingeri* Burgeon, 1928	Lebiinae	Sp	0	0	1	0	0	0	0	0	0	0	0	0	1
*Graphipterustristis* Klug, 1853	Lebiinae		2	1	1	14	2	0	4	1	0	1	0	0	26
Anaulacus (Aephnidius) madagascariensis (Chaudoir, 1850)	Lebiinae		0	0	0	1	0	0	7	1	1	0	0	0	10
Tetragonoderus(s. str.)immaculatus La Ferté-Sénectère, 1853	Lebiinae	Sp	0	0	0	7	5	0	22	0	0	0	0	0	34
*Cymindoidearegularis* Basilewsky, 1961	Lebiinae	Sp	0	0	0	0	1	0	0	0	0	0	0	0	1
*Platytarustessellatus* (Dejean, 1831)	Lebiinae	G	0	0	0	1	0	0	0	0	0	0	0	0	1
*Apristuslatipennislatipennis* Chaudoir, 1878	Lebiinae	G	0	0	0	0	1	0	0	0	0	0	0	0	1
*Microlestesflavipesmicromys* Alluaud, 1918	Lebiinae		0	0	0	0	0	0	26	3	2	1	0	3	35
*Microlesteszambezianus* Mateu, 1960	Lebiinae		0	0	0	0	0	0	41	7	11	225	34	52	370
Mesolestes(s. str.)machadoi Mateu, 1965	Lebiinae	Sp	0	0	0	0	0	0	6	0	0	0	0	0	6
Mesolestes(s. str.)nigrocephalus Mateu, 1962	Lebiinae	Sp	0	0	5	0	0	1	10	2	0	0	0	0	18
*Mesolestes* sp.	Lebiinae		1	0	0	0	0	0	0	0	0	0	0	0	1
Singilis(s. str.)africaorientaliskenyacus Anichtchenko, 2016	Lebiinae	Sp	0	0	0	0	0	0	1	0	0	0	0	0	1
Planetes(s. str.)quadricollis Chaudoir, 1878	Dryptinae		0	0	0	0	0	1	0	0	0	0	0	0	1
*Galeritaangustipennis* Gerstaecker, 1867	Dryptinae		0	0	0	0	0	2	0	0	0	0	0	0	2
*Triaenogeniuscarinulatuscarinulatus* (Fairmaire, 1887)	Anthiinae		0	0	0	0	0	1	0	0	0	0	0	0	1
*Cypholobaalveolataranzanii* (Bertoloni, 1849)	Anthiinae		2	3	0	0	0	0	0	0	0	0	0	0	5
*Cypholobagraphipteroidesbilunata* (Boheman, 1860)	Anthiinae		0	0	7	1	1	3	4	0	7	0	0	0	23
*Cypholobarutata* (Péringuey, 1892)	Anthiinae		2	0	5	5	2	2	0	0	0	0	0	0	16
*Cypholobasemisuturatavassei* (Sternberg, 1907)	Anthiinae		0	0	1	0	0	0	0	0	0	0	0	0	1
*Eccoptopteramutilloidesmutilloides* (Bertoloni, 1857)	Anthiinae		1	1	0	0	0	1	0	0	0	0	0	0	3
Anthia (Termophilum) alternata Bates, 1878	Anthiinae		2	0	2	8	4	3	2	0	0	0	0	3	24
Anthia (Termophilum) burchelli petersi Klug, 1853	Anthiinae		0	0	0	0	0	1	2	1	1	0	0	0	5
Anthia (Termophilum) omoplata Lequien, 1832	Anthiinae		1	3	1	0	0	0	0	0	0	0	0	0	5
Anthia (Termophilum) fornasinii fornasinii Bertoloni, 1845	Anthiinae		3	0	1	0	0	0	1	0	0	0	0	0	5
Anthia(s. str.)circumscriptacircumscripta Klug, 1853	Anthiinae		0	0	2	0	1	0	4	2	0	0	0	0	9

**Table 3.** Overall species richness and abundance of Caraboidea in the study habitats for the three sampling periods (T1: 25 October-5 November; T2: 5-15 November; T3: 15-25 November 2019). Number of collected pitfall traps (out of 75) is indicated.

**Habitat**	**Sampling period**	**Number of collected pitfalls**	**Abundance**	**Species richness**
Miombo Tropical Forest	T1	72	28	16
	T2	75	31	11
	T3	73	133	27
Mixed Dry Forest	T1	71	53	16
	T2	71	40	21
	T3	60	242	33
Transitional Forest	T1	66	344	25
	T2	64	169	16
	T3	64	201	36
Grassland	T1	69	246	14
	T2	68	47	8
	T3	32	250	29
